# An Eye-Tracking Study of Sketch Processing: Evidence From Russian

**DOI:** 10.3389/fpsyg.2020.00297

**Published:** 2020-03-02

**Authors:** Tatiana E. Petrova, Elena I. Riekhakaynen, Valentina S. Bratash

**Affiliations:** ^1^Laboratory for Cognitive Studies, Saint-Petersburg State University, Saint Petersburg, Russia; ^2^Department of General Linguistics, Saint-Petersburg State University, Saint Petersburg, Russia; ^3^Department of Education, Saint-Petersburg State University, Saint Petersburg, Russia

**Keywords:** eye-tracking, sketchnoting, text comprehension, text processing, Russian

## Abstract

This study investigates the online process of reading and analyzing of sketchnotes (visual notes containing a handwritten text and drawings) on Russian language material. Using the eye-tracking method, we compared the processing of different types of sketchnotes [“path” (trajectory), linear, and radial] and the processing of a verbal text. Biographies of Russian writers were used as the material. In a preliminary experiment, we asked 89 college students to read the biographies and to evaluate each text or sketch using five scales (from −2 to +2). The best example for each of three formats of sketchnotes and a verbal text was chosen. In the main experiment, 21 secondary school students examined four different biographies in four different formats (three sketchnotes and a verbal text), answered to the factual and analytical questions to these texts and estimated the difficulty of each text. We measured the total dwell time, the total fixation count, the average fixation duration for each stimulus as well as for separate zones inside the sketches including verbal and non-verbal information. Our results show that readers process the information better and faster while reading sketchnotes than a verbal text. In the trajectory sketchnotes, the readers followed the order of elements aimed by the author of the sketchnotes better than in the radial and linear sketchnotes. The analysis of participants’ eye movements while processing the stimuli made it possible to propose several recommendations for creating effective sketchnotes.

## Introduction

Nowadays, there is a growing trend toward the use of visual information in various spheres of life (psychology, education, marketing, etc.). Texts containing two non-homogeneous parts – verbal and non-verbal semiotic resources (or “modes”) – have become an integral part of communication. The studies of infographics (graphic visual representation of information), sketchnoting (visual notes including a handwritten text and drawings), advertising copies, multimedia courses integrating the verbal and non-verbal elements are of particular relevance.

The polycode text analysis is traditionally based on the Dual Cording Theory (DCT) (Paivio, 1971, 1986). The theory assumes that there are two distinct cognitive systems: one for processing verbal units and the other one (imagery) for dealing with non-verbal objects/events. Paivio (2006) indicates that the information is represented in the memory by a text and a corresponding illustration, not just by a text. It is assumed that the information in a polycode text is double-decoded: the concept of an image is “superimposed” on the concept of a verbal text, the interaction of these two concepts leads to the creation of a general concept (meaning) of the text (Telminov, 2009; Fernández-Fontecha et al., 2018). Independent parts of a polycode text interact and create a “holistic experience,” the combination of the visual language with the written language.

In many studies, the influence of visual components on the comprehension of the whole polycode text is evaluated by offline tests (questionnaires, scales, etc.) (Cohn, 2016). However, these methods can only measure the result of the comprehension process. Thus, the identification of the particular elements which influence different stages of the process is difficult. Eye tracking techniques provide online information about learners’ behavior during text reading. As Rayner (1998) points out, by using eye tracking, one can study reading as a process, instead of “a mere end-result.” During the last 20 years, a lot of empirical and experimental evidence on online processing of polycode texts (including comics and visual narratives) appeared. One of the paradigms is called Visual Language Theory (VLT) which describes how visual lexical items are read taking into consideration the structure of polycode samples and trying to develop the “narrative grammar of sequential images” (Cohn, 2018). This approach argues that verbal and non-verbal components operate in parallel as interfering structures.

Polycode texts are regarded as a new type of texts used in education (Kazakova, 2016). They have become a crucial part of teaching in a wide range of academic and practical disciplines (Altieri, 2017; Chandler, 2017). The educational aspects of the polycode text processing are considered among others in the Cognitive Theory of Multimedia Learning (CTML) (Mayer, 2009). This theory assumes that the combination of verbal information and pictures makes it easier for learners to understand and memorize a text. While studying the processing of verbal and non-verbal information, Levie and Lentz (1982) concluded that the information supported by both a text and a picture is acquired much better. The more switches there are between a text and an image while reading a polycode text, the better a reader understands the material (e.g. Mason et al., 2013; Scheiter and Eitel, 2015). It has been shown that if students do not pay enough attention to the pictures and focus mainly on the text zones or untimely correlate verbal and visual information, the effectiveness of training falls significantly (Hannus and Hyönä, 1999; Schwonke et al., 2009; Schmidt-Weigand et al., 2010; Cromley et al., 2013a, b; Mason et al., 2013; Renkl and Scheiter, 2017). Moreno and Mayer (2002) and Johnson and Mayer (2012) tried to solve this problem by means of additional instructions. Ozcelik et al. (2009) and Scheiter and Eitel (2015) used spatial-color schemes reducing the distance between the text and the picture or highlighting the corresponding verbal and non-verbal elements in one color. However, such tools were shown to be effective only for poorly prepared students, but do not influence the results of students with a higher level of training (Kalyuga et al., 2003; Sweller et al., 2003; Kalyuga, 2007; Richter et al., 2017).

This research is conducted within the CTML and is aimed to study the processing of sketchnoting (or visual notes) as an example of a polycode (multimodal) text. As far as we know, sketchnotes have never become the object of a psycholinguistic research using online methods, although they seem to be worthwhile both for learning how we process multimodal information and for educational purposes as a new type of data compression. Sketchnoting combines various ways of presenting information and includes such uncommon for other types of polycode texts elements as hand-drawn typography, handwritten (not printed) texts, and many different visual components: drawings, arrows, lines, and dots (Rohde, 2013). Moreover, there are several distinct types of the organization of the material in sketchnotes. There are seven types of sketch structures: (1) path (trajectory; with arrows helping to navigate the text), (2) linear (information and visual components are arranged as in a normal verbal text – lines going from the left to the right), (3) radial (the main idea of the text is in the middle of the list surrounded by other text elements), (4) vertical (text elements are organized vertically: from the top to the bottom of the page), (5) modular (each piece of information forms a separate block), (6) skyscrapers (the information is organized in several vertically stretched rectangles), and (7) popcorn (with random arrangement of all blocks of information) (Rohde, 2013: 90) (see the layouts of all sketch structures in Supplementary Figure 1). Thus, we can compare how different structures are processed, explore the impact of a sketch type on the navigation decisions, and find out the most efficient sketch structure for retrieving the information. The aim of our study was to compare the processing of sketchnotes and a verbal text and to choose the best type of sketchnotes for transferring the information to a reader.

## Preliminary Experiment

### Goal

The goal of the preliminary experiment was to choose the stimuli for the main experiment, i.e. the sketchnotes of different structures and a verbal text that are evaluated as the most attractive (interesting, informative, good-structured, etc.) by school children – native speakers of Russian.

### Material

We chose the biographies of four Russian poets for our study. A biography is usually a stereotyped text with a standard structure (including such common information as the years of life, the place of birth, education, some information about the family, profession, interests, the main stages of life, etc.). Biographies are often used while studying literature at school.

The poets were as follows: O. Mandelshtam, M. Voloshin, Z. Gippius, and I. Severyanin. All of them lived in the first half of the 20th century and their poems are not included in the obligatory school program in Russia. Thus, we can assume that the background of our participants who were school children did not influence significantly their performance in the experiments as most probably they were not familiar with the biographies we had chosen for our study.

The initial biographies were in verbal format (plain texts) and taken from one and the same resource (guide on literature). All the texts were of the same size and comparable level of readability^1^ (Supplementary Table 1). To get the stimuli, we converted all biographies into three main sketch formats (that contain the features of all other types of sketchnotes): trajectory, linear, and radial using the guidelines provided in Rohde (2013). Thus, the material of the experiment consisted of 4 different verbal texts and 12 sketchnotes – three formats for each of four different biographies^2^. The readability level in all types of the sketchnotes was lower than in the plain texts.

### Procedure and Participants

We asked 89 Russian school children (45 girls) to read the biographies and to evaluate each text or sketch using five scales (from −2 to +2 each): non-informative – informative, difficult to understand – easy to understand, not interesting – interesting, difficult to retell – easy to retell, bad structure – good structure. We used the Latin Square design. Every participant read four different biographies each of them presented either as a verbal text or in one of three sketch formats. Thus, every participant saw each type of the text and each biography only once. All the stimuli were presented in randomized order. The experiment lasted around 20 min for each participant.

### Results

For each of 16 stimuli, we summed up the scores from all five scales for each participant and compared these aggregate scores for different formats of presentation of one and the same biography (using ANOVA and the Kruskal–Wallis test for independent samples according to the type of data distribution). We found the factor of the format of presentation to be significant for three out of four biographies. To reveal the best format for each of these three biographies we performed the unpaired two-sample *t*-tests for each pair of formats within each biography. Surprisingly, for all four biographies, we got quite high aggregate scores for the verbal format. The sketchnotes that turned out to be significantly different from the verbal text are marked with an asterisk (^∗^) on Supplementary Figures 2–5. We did not find the significant difference between the verbal text and the trajectory sketchnotes for any of the biographies.

For the main experiment, we had to choose four different formats of presentation from the preliminary experiment (linear sketchnotes, radial sketchnotes, trajectory sketchnotes, and a verbal text). As we planned to show all four formats for every participant in the main experiment, we could choose only one stimulus for each biography. Thus, taking into consideration this condition, we were choosing among the stimuli with the highest aggregate scores for each biography and finally got the following set of stimuli: (1) the biography of Z. Gippius – the verbal text; (2) the biography of I. Severyanin – the trajectory sketchnotes; (3) the biography of O. Mandelshtam – the radial sketchnotes; and (4) the biography of M. Voloshin – the linear sketchnotes. The text parts of the sketchnotes 2–4 were of a comparable readability level (Supplementary Table 2) and had the equal number of pictures.

## Main Experiment

### Hypothesis

The hypothesis of the experiment was that readers process different text formats differently, trajectory sketchnotes being easier to process and understand than other types of sketchnotes and a verbal text.

### Participants

Twenty-one native speakers of Russian (secondary school children, 13–18 years old, 11 girls), who had not participated in the preliminary experiment, took part in the main experiment on voluntary basis. All subjects had normal or corrected to normal vision.

### Procedure

We conducted an eye-tracking experiment. We used a SR Eyelink 1000 plus eye tracker (SR Research Ltd., ON, Canada) with a head holder (“desktop mode” configuration) and 27′ LCD monitor (Acer v276hl) with a refresh rate of 60 Hz (screen resolution 1920 × 1080) to record the eye-movements of the participants. Viewing distance was 87 cm. It differs from the recommended eye-to-monitor distance for Eyelink 1000+, but it was the only way to place the monitor in the given conditions. We conducted several pilot trials and revealed that a participant could see all the letters and pictures of the stimuli at this distance and the nine-point calibration and validation were successful. The average error level during calibration was <0.5°; the threshold was 1°. Although viewing was binocular, we recorded participants’ dominant eye. All but two of the participants had the right dominant eye. We used SR Research Experiment Builder to create and run the experiment and EyeLink Data Viewer to analyze the results.

After successful calibration and validation each subject received an instruction to examine four different biographies sequentially presented on the computer screen and be ready to answer the questions after each text or sketch. All biographies were presented on the computer screen for 5 min. The participants were free to press the spacebar button if they were ready to answer the questions earlier than after 5 min. For each biography, we prepared four factual questions, three questions revealing the general comprehension of the sketch or the text and one rating scale question for estimating whether the text was difficult or easy to understand (from −2 = very easy to +2 = very difficult). The list of questions for each sketch and a verbal text can be found here: https://drive.google.com/drive/folders/1xgpKcymbzI28bYoy3QpGINvoQDcQzcGz?usp=sharing. The participants answered orally. One of the experimenters marked correct answers in a special paper form. We also used a digital voice recorder Olympus WS-65OS to record the participants’ responses to be able to revise the data. We used drift correction before presenting each text or sketch and if it turned out to be unsuccessful, we performed recalibration. The experiment lasted for about 40 min (including the calibration and recalibration period).

The experiment was conducted *in July 2018* at the Educational Centre “Sirius” (Sochi, Russia) in accordance with the Declaration of Helsinki and the existing Russian and international regulations concerning ethics in research. It was approved by the Ethics Committee of Saint-Petersburg State University in June 2018. As the participants were under 18 years old, we obtained written informed consents for their participation in the experiment from their parents.

### Measures

We considered several global eye movement measures traditionally used for studying polycode text processing (dwell time, total fixation count, average fixation duration). As the aim of the research was to compare the processing of different types of the sketchnotes, we also calculated the number of deviations from the trajectory aimed by the author of the sketchnotes while each participant processed every sketch. We also segmented all sketchnotes into interest areas, i.e. verbal and non-verbal elements of the sketch, and analyzed interest area dwell time, interest area first run dwell time, interest area fixation count for each verbal and non-verbal zone of the texts in order to compare the processing of different structural elements of the sketchnotes. The number of correct answers to the factual and analytical questions and the subjective difficulty of different stimuli were also analyzed.

### Results

Due to some technical problems, we did not manage to record the eye-movements of three participants while processing one of the formats (twice the verbal text and once the trajectory sketchnotes) and the eye-movements of one more participant while processing two formats (the verbal text and the linear sketchnotes). Thus, when we compared the processing of different formats by one and the same participant, we excluded the results of these four participants.

The Friedman test showed the influence of the factor “Format type” on the parameters “Dwell time” [χ^2^(3) = 19.24, *p* < 0.001], “Total fixation count” [χ^2^(3) = 23.61, *p* < 0.001], and “Average fixation duration” [χ^2^(3) = 12.88, *p* = 0.005]. According to Conover’s *post hoc* tests, sketchnotes of any format were read significantly more quickly and with a smaller number of fixations than the text whereas the processing of different types of sketchnotes did not differ significantly (see Supplementary Tables 3, 4 and Supplementary Figures 6, 7, respectively). The difference in the average fixation duration is not that clear-cut. There is no significant difference between the average fixation duration for the trajectory and radial sketchnotes (*p* = 0.882), the linear and radial sketchnotes (*p* = 0.059), and the linear sketchnotes and the text (*p* = 0.186) whereas in all other pairs we did find significant differences. The mean fixation duration for the text is shorter than for any type of the sketchnotes, but the results not of all the participants follow this tendency.

While reading the trajectory sketchnotes the participants significantly more often (*p* = 0.019 in the Binomial test) followed the order of reading the sketch elements aimed by the author of the sketchnotes than diverged from it (we considered that the participant diverged from the aimed trajectory if there were three or more deviations) (Supplementary Table 5). While processing the radial sketchnotes, there were more participants who followed the order of reading than those who did not, but the difference was not statistically significant (*p* = 0.245). Only around 30% of the participants followed the order of the sketched elements aimed by the author while processing the linear sketchnotes (Supplementary Table 5).

Our results also revealed that all the sketchnotes were subjectively evaluated by the participants to be easier to understand than the verbal text (the median value Me = 2 and Me = 1, respectively). The participants answered correctly to significantly more questions after all sketchnotes than after the verbal text. The influence of this factor was shown by the Friedman test: χ^2^(3) = 18.26, *p* < 0.001; the Conover’s *post hoc* tests demonstrated the significant difference between the results for the text and for all types of the sketchnotes (Supplementary Table 6). The same was true if we compared the number of correct answers only to factual questions. For analytical questions, we got significantly better results for the linear and trajectory sketch, whereas for the radial sketch the distribution of correct and incorrect answers did not show statistically significant difference from the results for the verbal text (Supplementary Table 7).

The radial sketch (the biography of Mandelshtam; see Supplementary Figure 8) is of particular interest since it contains both horizontal and diagonal zones. We compared the processing of a horizontal zone (interest areas “Mtext_1_mood” and “Mtext_2_Pushkin” together) to the processing of a diagonal zone (“Mtext_6_epigramma_diagonal”) of the same size (containing equal number of symbols: 154 and 156, respectively) and revealed that the dwell time for the horizontal zone was significantly less than for the diagonal zone (*W* = 45, *p* = 0.024).

We compared the processing of zones containing verbal and non-verbal information in the linear sketch (the biography of Voloshin; see Supplementary Figure 9) as it was the only sketchnotes where there were several comparable pieces of information presented both in verbal and non-verbal format. These were the portraits of Russian poets and writers (“Bimage_2_Cvetaeva,” “Bimage_13_Beli,” “Bimage_14_Gorki”) and signs with their names (“Btext_12_Cvetaeva,” “Btext_13_Beli,” “Btext_14_Gorkij”). These zones of interest were of the same size and the same content. We revealed that the verbal components in all three image-text pairs were processed less quickly (Tsvetaeva – *W* = 176, *p* = 0.006; Belyj – 187, *p* < 0.001; Gor’kij – 156, *p* = 0.015). However, we didn’t find this effect for the portrait of the main hero. There was no significant difference between the processing of the portrait of Voloshin (“Bimage_1_partrait”) and the verbal zone with his name (“Btext_1_titel”) above it (*W* = 120, *p* = 0.596).

The average time spent on the title zones turned out to differ significantly in all three sketchnotes being the longest for the biography of Mandelshtam (the radial sketchnotes) and the shortest for the biography of Severyanin (the trajectory sketchnotes) [see Supplementary Figure 11, Supplementary Table 8, and the heat maps (Supplementary Figures 8–10)].

## Discussion

In our study, we found that the processing of any type of sketchnotes where verbal information is combined with non-verbal differs significantly from the processing of the verbal text. These results correlate with the previous studies that showed that an image and a written text presented together can contribute to a better understanding of the information than if they are presented separately (Schnotz, 2005) and with the CTML (Mayer, 2009) that assumes that a multimodal text is an effective form of learning as it implicates switching of attention between a text and an image and establishes links between the two elements. The so-called multimedia effect helps to integrate the new information in the cognitive system and to remember it.

As it was shown in numerous eye-tracking studies, a text is read according to the *F*-shaped scanning pattern which is characterized by many fixations concentrated at the top-left part of the screen (Pernice, 2017). We got the same results for the processing of the biography presented as a verbal text. There were more gazes on the first lines than on the subsequent ones. The first several words on the left of each line received more fixations than subsequent words in the same line (Supplementary Figure 12). For all the sketchnotes we analyzed, the reading patterns were usually text-directed. This result correlates with other studies of polycode texts that showed that the text zones receive more attention than the picture zones (Rayner et al., 2001; Petrova and Riekhakaynen, 2019). Lee and Wu (2017) have also shown that a picture or a geometric figure attracts less reader’s attention than a text in the process of scanning math texts. Although the sketchnotes we analyzed represented three different types of information organization, we did not find any significant differences in the time of their processing and, the number of fixations, subjective evaluation, and the number of correct answers to the after-the-text questions. However, while processing the trajectory sketchnotes the participants followed the order of reading aimed by the author better than while processing the linear and radial sketchnotes. We presume that, despite the fact that the participants did not pay much attention to the zones with small arrows that were numerous in the trajectory sketch (there were few fixations on them), these arrows helped not to deviate. We also did find some differences in the average fixation duration between the sketchnotes we analyzed. These results require further consideration, but we presume that the factors influencing the average fixation duration include the font size, the number of pictures in the texts, as well as the individual strategies of participants.

Our results also allow to discuss some basic principles of the polycode text structure. Although the pictures usually attract less attention than the verbal text containing the same information, the portraits of the main characters are normally scanned more attentively than other images. This finding is close to some recent face recognition eye-tracking studies and recommendations to use portraits and pictures of a person’s face in order to increase reader’s attention to a website (Patel, 2014) and banner advertisements (Sajjacholapunt and Ball, 2014). At the same time, the results we got on how a reader scan the titles of the sketchnotes do not correspond to the prior studies that showed that readers paid more attention to headings (e.g. Hyönä et al., 2002; Hyönä and Lorch, 2004; Lemarié et al., 2012) and found them useful when reading a text (Hartley and Trueman, 1985; Yussen et al., 1993), encoding the topic-comment structure of a text and recalling the text content (Lorch and Lorch, 1995). It was revealed that different types of headings influence the process of searching the text and the sequence of examination of text elements (Klusewitz and Lorch, 2000). Our results show that the participants do not pay much attention to the title zones. However, we still believe that the headings are helpful for finding the target information in the text and can be used to guide the process of examining the text or sketch. According to the results we received, to attract more attention the title in a polycode text should be somehow included in the overall structure of the sketchnotes or should be placed in non-standard way.

## Conclusion

Reading is a complex task that depends on many different cognitive processes. Numerous experiments have shown that text understanding is a complex multistep process. The comprehension of a written text includes – among others – the recognition and pattern analysis of letters, graphics, and structural components. Recent cognitive-orientated research shows that the text type is among the readability categories. The aim of the present study was to reveal whether a sketch or a verbal text is easier to process and better to use for retrieving the essential information.

Eye-tracking studies of the processing of Russian texts are not numerous. They are mainly focused on the recognition of a regular verbal text (Bezrukikh and Ivanov, 2013, 2014, 2015; Kornev et al., 2014; Petrova, 2016; Korneev et al., 2017a, b). There is only one eye-tracking research on Russian (Petrova and Riekhakaynen, 2019) in which the processing of a polycode text, namely infographics, has been studied. It was one of the first steps to reveal how readers integrate text–figure information when reading and understanding infographics.

The results of the present study have shown that a sketch of any format is read faster than a verbal text. It is worth mentioning that the percentages of correct answers to the after-the-text questions are normally higher after processing sketchnotes than after reading a verbal text. The trajectory (path) seems to be the most efficient type of sketchnoting because it clearly shows a reader the order of reading aimed by the author of the sketchnotes.

The analysis of participants’ eye movements while processing the stimuli allowed us to propose a number of recommendations for creating sketchnotes: (1) diagonal position of the text is not efficient because such zones are read significantly slower than the zones where the text is arranged horizontally; (2) it is better to control the reader’s attention with the arrows as they show the order of acquiring the information presumed by the author of a sketch and thus help to learn the text faster; and (3) it is important to duplicate the information from the title somewhere inside the sketchnotes or to integrate the title into the sketch to attract reader’s attention to it.

We suppose that visual notes can be a functional alternative of a traditional verbal summary and this format can diversify the educational process. It is possible to recommend using sketchnoting as an alternative way of processing large blocks of information, when a reader can decide himself what type of summary to choose. The data obtained open perspectives for further investigation of the reading process, means of resolving ambiguity in the different text types, and the relationship between verbal and non-verbal parts of the text.

## Data Availability Statement

All datasets generated for this study are included in the article/Supplementary Material.

## Ethics Statement

The experiment was conducted in July 2018 at the Educational Centre “Sirius” (Sochi, Russia) in accordance with the Declaration of Helsinki and the existing Russian and international regulations concerning ethics in research. It was approved by the Ethics Committee of Saint-Petersburg State University in June 2018. As the participants were under 18 years old, we obtained written informed consents for their participation in the experiment from their parents.

## Author Contributions

TP: main idea, data collection for the experiments, introduction, and discussion. ER: the eye-tracking experiment and analysis of the results, figures, and tables. VB: choosing the stimuli, data collection, and creating sketchnotes. All authors contributed to the research and to the manuscript, and agreed to be accountable for the content of the work.

## Conflict of Interest

The authors declare that the research was conducted in the absence of any commercial or financial relationships that could be construed as a potential conflict of interest.

## Footnotes

1 Checked via http://readability.io/.

2 The data are available at: https://drive.google.com/drive/folders/1xgpKcymbzI28bYoy3QpGINvoQDcQzcGz?usp=sharing
