# Supplementary material for: An Eye-Tracking Study of Sketch Processing: Evidence From Russian
Source: Front Psychol. 2020 Mar 2;11:297. doi: 10.3389/fpsyg.2020.00297 (PMC7061926; doi:10.3389/fpsyg.2020.00297)
Supplement: Supplementary file 1 [file Table_1.DOCX]

Supplementary Material

## Supplementary Figures

##
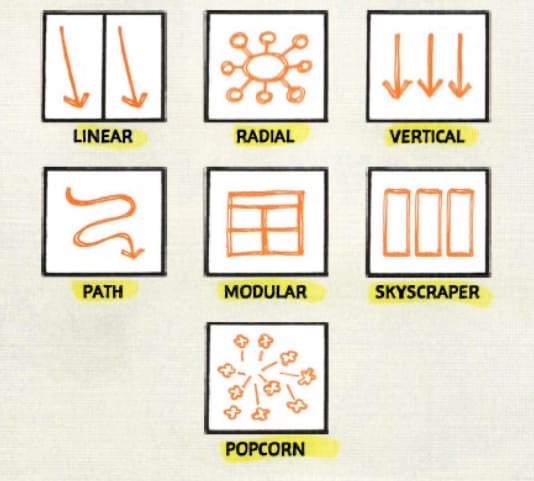


## Supplementary Figure 1. The layout of all sketch structures (Rodhe, 2013: 90).


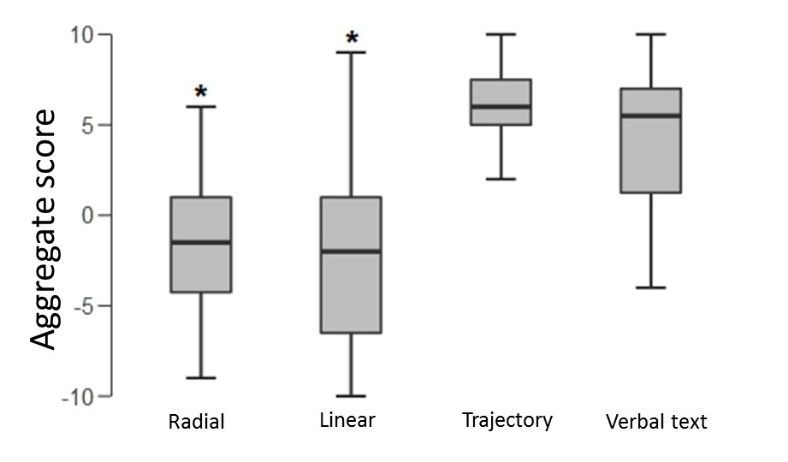


**Supplementary Figure 2.** The biography of Z. Gippius: the boxplots show the distribution of the aggregate scores received from all the participants for each type of the presentation (three types of sketchnotes or verbal text). For every participant, we summed up the scores he marked on each of five scales (see section 2.2. Material). As each scale was from -2 to +2, the maximum aggregate score could be +10 and the minimum aggregate score could be -10.


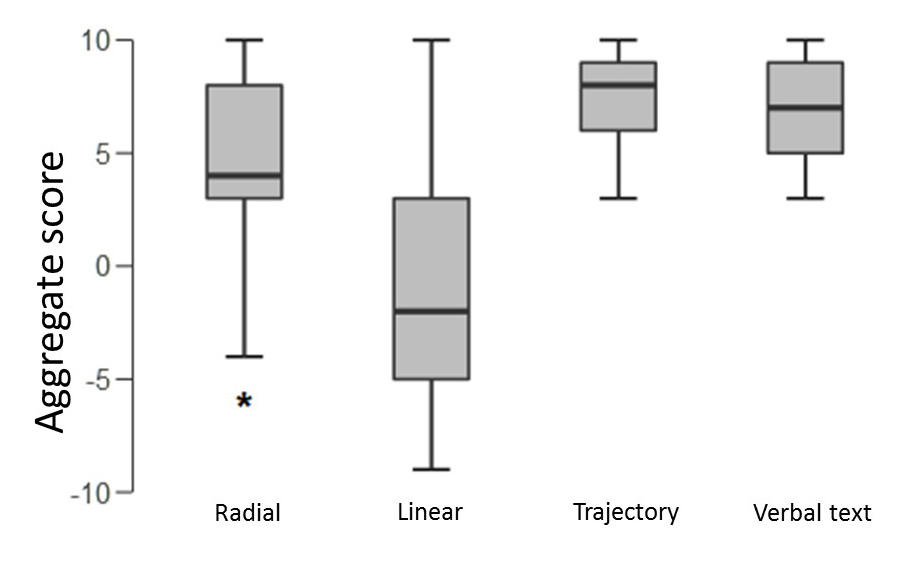

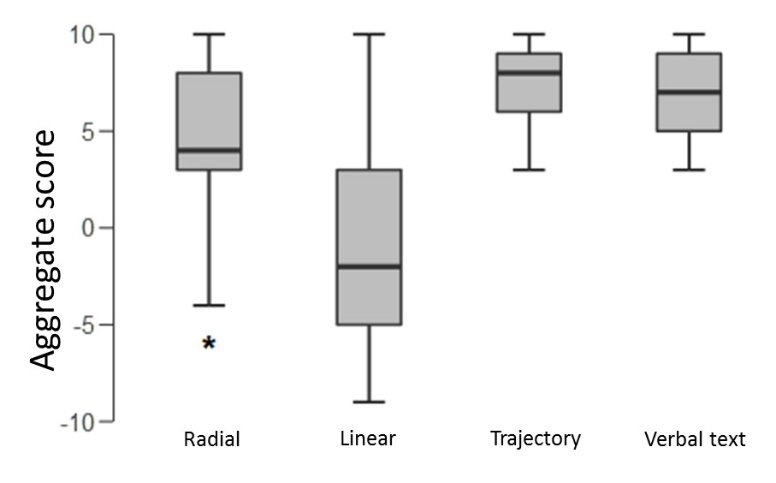


**Supplementary Figure 3.** The biography of I. Severyanin: the boxplots show the distribution of the aggregate scores received from all the participants for each type of the presentation (three types of sketchnotes or verbal text). For every participant, we summed up the scores he marked on each of five scales (see section 2.2. Material). As each scale was from -2 to +2, the maximum aggregate score could be +10 and the minimum aggregate score could be -10.


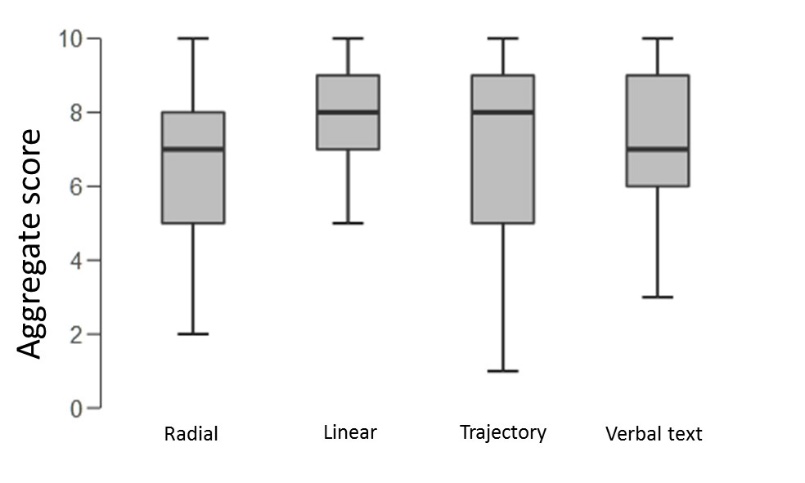


**Supplementary Figure 4.** The biography of O. Mandelshtam: the boxplots show the distribution of the aggregate scores received from all the participants for each type of the presentation (three types of sketchnotes or verbal text). For every participant, we summed up the scores he marked on each of five scales (see section 2.2. Material). As each scale was from -2 to +2, the maximum aggregate score could be +10 and the minimum aggregate score could be -10.


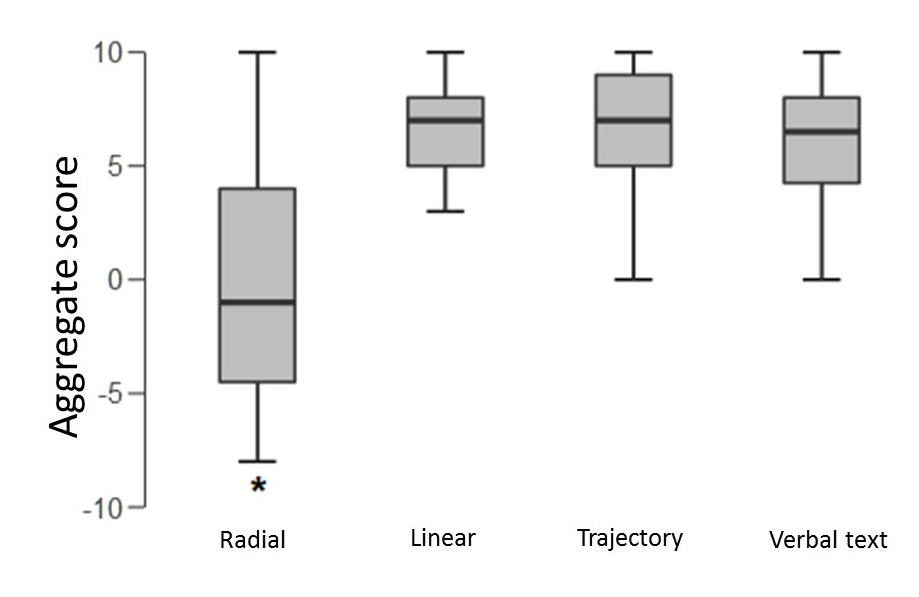


**Supplementary Figure 5.** The biography of M. Voloshin: the boxplots show the distribution of the aggregate scores received from all the participants for each type of the presentation (three types of sketchnotes or verbal text). For every participant, we summed up the scores he marked on each of five scales (see section 2.2. Material). As each scale was from -2 to +2, the maximum aggregate score could be +10 and the minimum aggregate score could be -10.


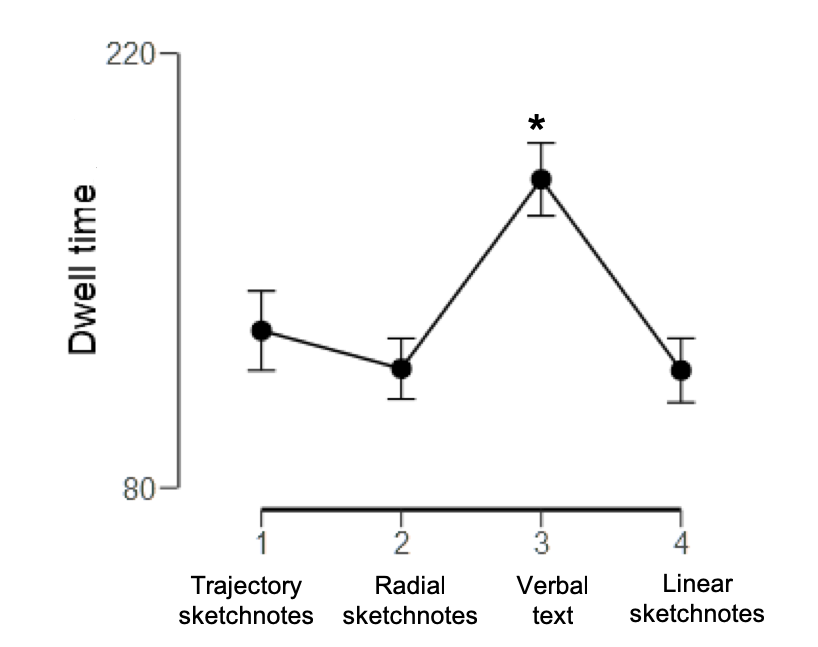


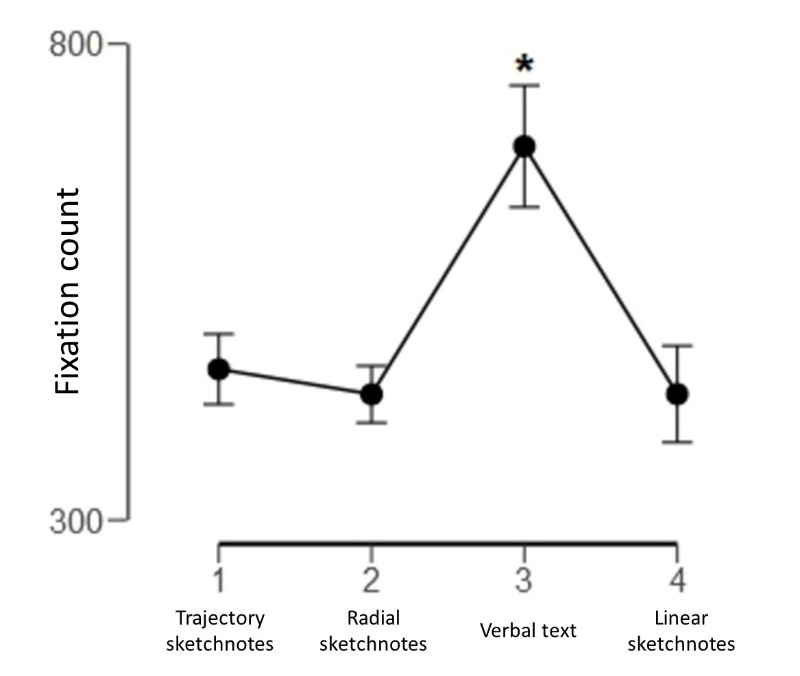
**Supplementary Figure 6.** The average dwell time (in seconds) for each sketch type and for the verbal text in the Main Experiment.

**Supplementary Figure 7.** The average number of fixations on each sketch type and on the verbal text in the Main Experiment.


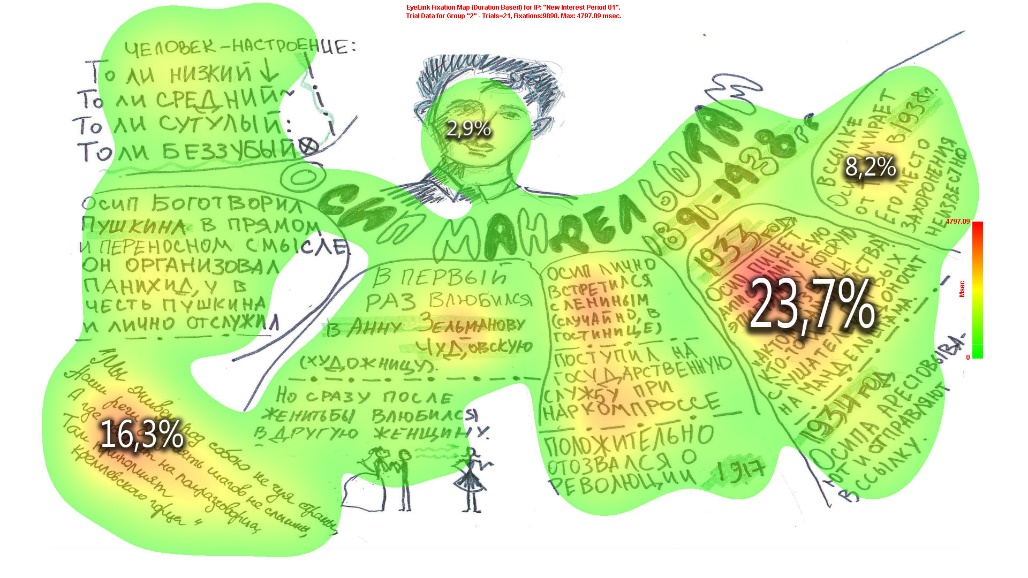


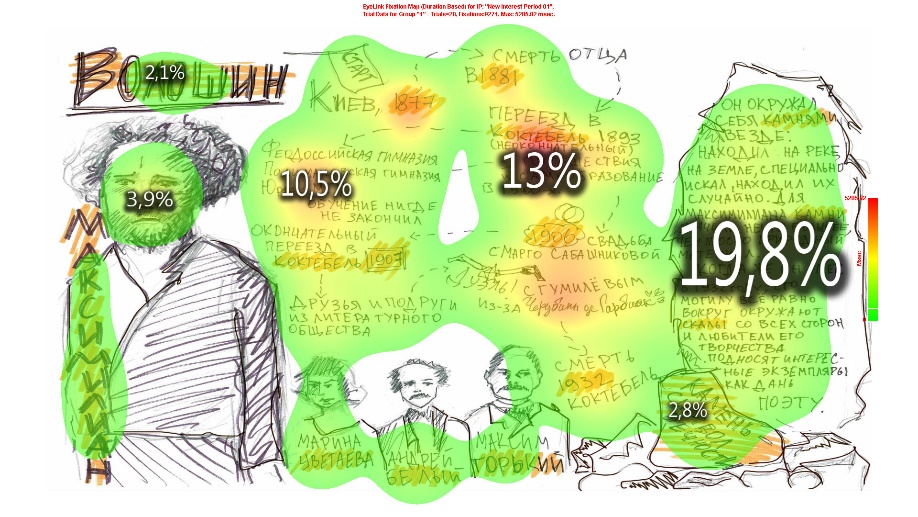
**Supplementary Figure 8.** The heat map for the radial sketchnotes.

**Supplementary Figure 9.** The heat map for the linear sketchnotes.


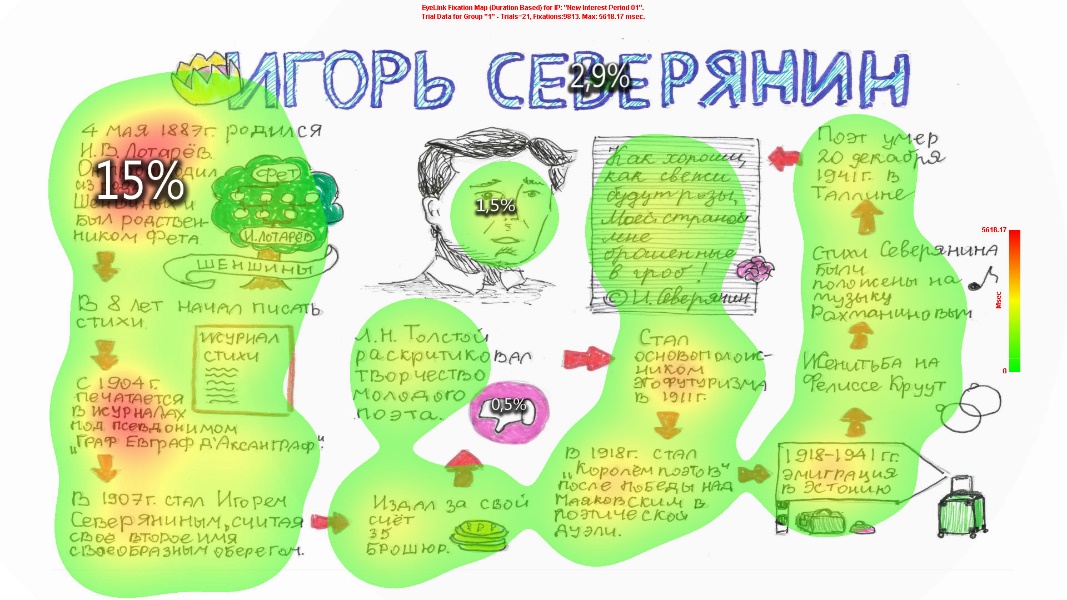


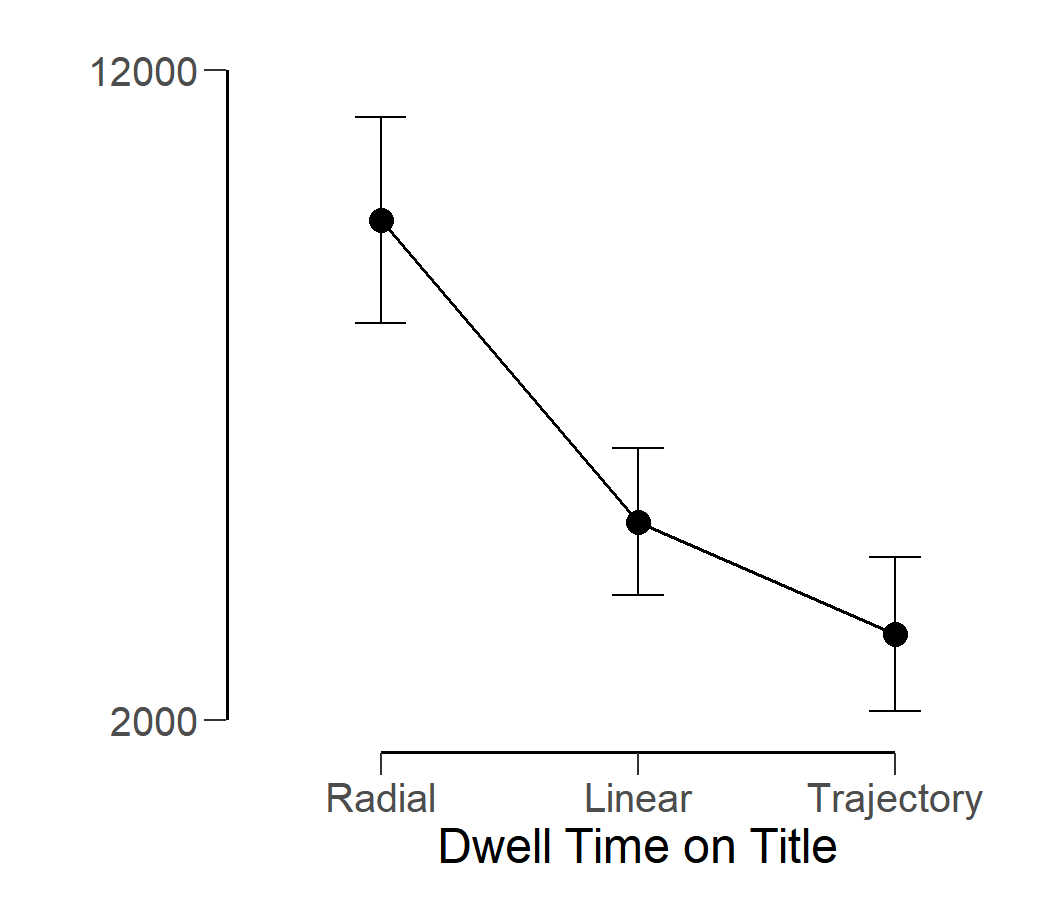
**Supplementary Figure 10.** The heat map for the trajectory sketchnotes.

## Supplementary Figure 11. Average dwell time on the titles of the sketchnotes.

##
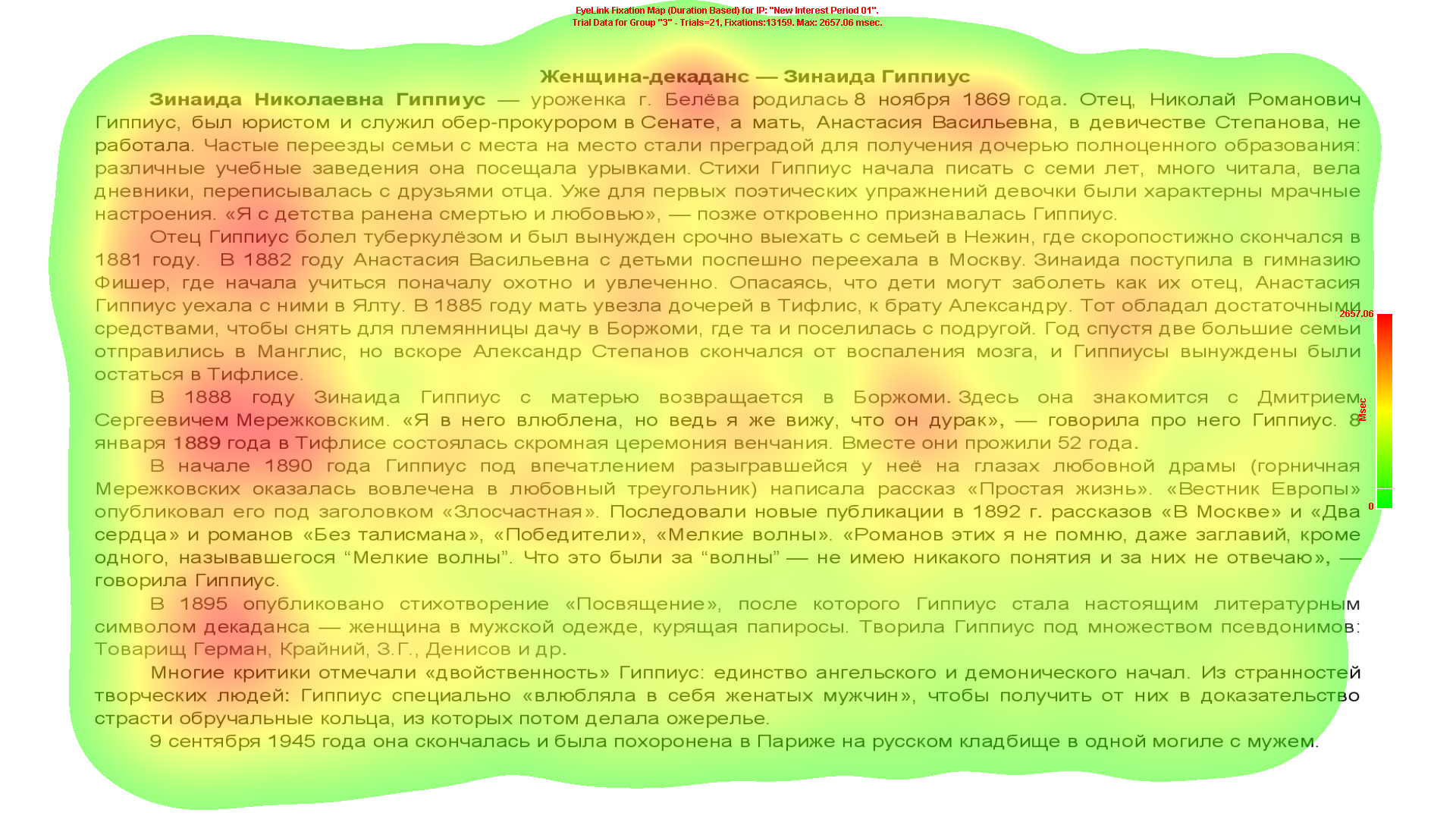


## Supplementary Figure 12. The heat map for the verbal text.

## Supplementary Tables

**Table 1.** Parameters of Texts in the Preliminary Experiment

|  | **SMOG Readability Score** | **Age of the Intended Audience (y.o.)** | **Length of the Text**  **(in symbols)** |
| --- | --- | --- | --- |
| Voloshin | 9.32 | 12-14 | 2670 |
| Severyanin | 9.47 | 12-14 | 2880 |
| Gippius | 9.17 | 12-14 | 2842 |
| Mandelshtam | 9.16 | 12-14 | 2662 |

**Table 2.** Parameters of Stimuli in the Main Experiment

|  | **SMOG Readability Score** | **Age of the Intended Audience (y.o.)** | **Length of the Text**  **(in symbols)** | **Length of the Text**  **(in words)** |
| --- | --- | --- | --- | --- |
| Linear sketch  (Voloshin’s biography) | 6.01 | 9-11 | 610 | 117 |
| Trajectory sketch (Severyanin’s biography) | 5.43 | 9-11 | 631 | 115 |
| Text format (Gippius’s biography) | 9.17 | 12-14 | 2461 | 410 |
| Radial sketch  (Mandelshtam’s biography) | 6.9 | 12-14 | 641 | 109 |

**Table 3.** Conover's Post Hoc Comparisons for Dwell Time Measure

|  | | | | | | | | | | | | | | |
| --- | --- | --- | --- | --- | --- | --- | --- | --- | --- | --- | --- | --- | --- | --- |
|  | |  | | **T-Stat** | | **df** | | **W _i_** | | **W _j_** | | **p** | |  |
| Trajectory |  | Radial |  | 1.306 |  | 48 |  | 41.00 |  | 33.00 |  | 0.198 |  |  |
|  |  | Text |  | 3.429 |  | 48 |  | 41.00 |  | 62.00 |  | 0.001 |  |  |
|  |  | Linear |  | 1.143 |  | 48 |  | 41.00 |  | 34.00 |  | 0.259 |  |  |
| Radial |  | Text |  | 4.736 |  | 48 |  | 33.00 |  | 62.00 |  | < .001 |  |  |
|  |  | Linear |  | 0.163 |  | 48 |  | 33.00 |  | 34.00 |  | 0.871 |  |  |
| Text |  | Linear |  | 4.572 |  | 48 |  | 62.00 |  | 34.00 |  | < .001 |  |  |

**Table 4.** Conover's Post Hoc Comparisons for Number of Fixations Measure

|  | |  | | **T-Stat** | | **df** | | **W _i_** | | **W _j_** | | **p** | |  |
| --- | --- | --- | --- | --- | --- | --- | --- | --- | --- | --- | --- | --- | --- | --- |
| Trajectory |  | Radial |  | 1.759 |  | 48 |  | 41.00 |  | 31.00 |  | 0.085 |  |  |
|  |  | Text |  | 4.045 |  | 48 |  | 41.00 |  | 64.00 |  | < .001 |  |  |
|  |  | Linear |  | 1.231 |  | 48 |  | 41.00 |  | 34.00 |  | 0.224 |  |  |
| Radial |  | text |  | 5.803 |  | 48 |  | 31.00 |  | 64.00 |  | < .001 |  |  |
|  |  | Linear |  | 0.528 |  | 48 |  | 31.00 |  | 34.00 |  | 0.600 |  |  |
| Text |  | Linear |  | 5.276 |  | 48 |  | 64.00 |  | 34.00 |  | < .001 |  |  |
|  | | | | | | | | | | | | | | |

| **Stimulus** | **Followed the aimed trajectory** | **Diverged from the aimed trajectory** | **P-value for**  **Binomial test** |
| --- | --- | --- | --- |
| Linear sketch  (Voloshin’s biography) | 6 | 14 | p=0.115 |
| Radial sketch  (Mandelshtam’s biography) | 14 | 7 | p=0.245 |
| Trajectory sketch (Severyanin’s biography) | 15 | 4 | p=0.019 |

**Table 5.** Number of participants following and not following the aimed trajectory of the sketchnotes

| **Table 6.** Conover's Post Hoc Comparisons for the Number of Correct Answers to the Questions | | | | | | | | | | | | | | | |
| --- | --- | --- | --- | --- | --- | --- | --- | --- | --- | --- | --- | --- | --- | --- | --- |
|  | |  | | **T-Stat** | | **df** | | **W _i_** | | **W _j_** | | **p** | |  |  |
| Text |  | Trajectory |  | 4.425 |  | 57 |  | 32.00 |  | 60.50 |  | < .001 |  |  |  |
|  |  | Linear |  | 4.192 |  | 57 |  | 32.00 |  | 59.00 |  | < .001 |  |  |  |
|  |  | Radial |  | 2.562 |  | 57 |  | 32.00 |  | 48.50 |  | 0.013 |  |  |  |
| Trajectory |  | Linear |  | 0.233 |  | 57 |  | 60.50 |  | 59.00 |  | 0.817 |  |  |  |
|  |  | Radial |  | 1.863 |  | 57 |  | 60.50 |  | 48.50 |  | 0.068 |  |  |  |
| Linear |  | Radial |  | 1.630 |  | 57 |  | 59.00 |  | 48.50 |  | 0.109 |  |  |  |
|  | | | | | | | | | | | | | | | |

**Table 7.** Percentage of Correct Answers to the After-the-Text Questions

| **Stimulus** | **Factual questions** | **Analytical questions** |
| --- | --- | --- |
| Linear sketch (Voloshin’s biography) | 85 % | 87 % |
| Radial sketch (Mandelshtam’s biography) | 82 % | 75 % |
| Trajectory sketch (Severyanin’s biography) | 83 % | 92 % |
| Text format (Gippius’s biography) | 61 % | 68 % |

| **Table 8.** Conover's Post Hoc Comparisons for the Dwell Time on the Titles of the Sketchnotes | | | | | | | | | | | | | | |
| --- | --- | --- | --- | --- | --- | --- | --- | --- | --- | --- | --- | --- | --- | --- |
|  | |  | | **T-Stat** | | **df** | | **W _i_** | | **W _j_** | | **p** | |  |
| Radial |  | Linear |  | 7.125 |  | 38 |  | 59.00 |  | 36.00 |  | < .001 |  |  |
|  |  | Trajectory |  | 10.532 |  | 38 |  | 59.00 |  | 25.00 |  | < .001 |  |  |
| Linear |  | Trajectory |  | 3.408 |  | 38 |  | 36.00 |  | 25.00 |  | 0.002 |  |  |
|  | | | | | | | | | | | | | | |
